# Zinc (II) and AIEgens: The “Clip Approach” for a Novel Fluorophore Family. A Review

**DOI:** 10.3390/molecules26144176

**Published:** 2021-07-09

**Authors:** Rosita Diana, Barbara Panunzi

**Affiliations:** Department of Agricultural Sciences, University of Naples Federico II, 80055 Portici, Italy; rosita.diana@unina.it

**Keywords:** AIE, zinc complex, fluorescence

## Abstract

Aggregation-induced emission (AIE) compounds display a photophysical phenomenon in which the aggregate state exhibits stronger emission than the isolated units. The common term of “AIEgens” was coined to describe compounds undergoing the AIE effect. Due to the recent interest in AIEgens, the search for novel hybrid organic–inorganic compounds with unique luminescence properties in the aggregate phase is a relevant goal. In this perspective, the abundant, inexpensive, and nontoxic d^10^ zinc cation offers unique opportunities for building AIE active fluorophores, sensing probes, and bioimaging tools. Considering the novelty of the topic, relevant examples collected in the last 5 years (2016–2021) through scientific production can be considered fully representative of the state-of-the-art. Starting from the simple phenomenological approach and considering different typological and chemical units and structures, we focused on zinc-based AIEgens offering synthetic novelty, research completeness, and relevant applications. A special section was devoted to Zn(II)-based AIEgens for living cell imaging as the novel technological frontier in biology and medicine.

## 1. Introduction

### 1.1. Activation of the Fluorescence Channel in AIEgens

A luminescent material is a material able to emit light in the process of returning from the electronic or vibrational excited state to the ground state after being excited by external energy. The term “luminescence” was introduced in 1888 by the physicist and historian Eilhard Wiedemann to describe light emission not simply related to an increase in temperature. Photo-induced luminescence or photoluminescence (PL), often referred simply as “luminescence”, is the light emission in the optical range of visible, ultraviolet, or infrared light. According to the mode of excitation and relaxation, luminescence can be classified into various types, including fluorescence and phosphorescence.

Specifically, “fluorescence” is a word coined from the mineral form of CaF_2_ known as fluorite. In 1819, E. D. Clarke reported that some crystals of green fluorite deeply emitted a blue colour under the illumination of a UV lamp. Contrary to phosphorescence, fluorescence is a form of photoluminescence in which the excitation–emission process occurs very quickly [1,2]. After a molecule absorbs energy from a light source and becomes excited, fluorescence occurs within nanoseconds. Upon excitation with electromagnetic energy at the correct wavelength, an electron in the fluorescent molecule is promoted to an upper level, and finally, the energy is released in the form of a photon (fluorescence emission) while the electron moves back down to the lower energy level. In most cases, fluorescence requires a longer excitation wavelength—and so lower energy—than the absorbed radiation. Therefore, fluorescence can occur when the absorbed radiation is in the ultraviolet (UV)-visible region of the spectrum, while the emitted light is in the visible region. Fluorescent dyes are compounds that strongly emit in the visible region so that they show a naked-eye-perceivable colour when exposed to a UV-visible light source [1,3,4,5,6].

Over the last century, the rapid development of molecular science moved to study the working mechanisms of fluorescence at the molecular level. Since 1800, fluorescent organic dyes highly emissive in diluted solutions were examined. In the middle of the nineteenth century, the chemist Adolf von Baeyer developed fluorescein as a synthetic organic material highly fluorescent in aqueous solutions in daylight. Early fluorescent materials found only rare applications, such as tracers for the detection of underground waterways or optical whitening agents in paper, textiles, and marking inks [7,8]. Even less attention was paid to the photoluminescence of aggregate molecules until the last century. The first report about the concentration effect on the fluorescence of aromatic compounds in solutions is due to J. B. Perrin, who in 1923 found that the photoluminescence of uranine (disodium salt form of fluorescein) decreased as the concentration increased. This fluorescence quenching effect at high concentrations was clarified in 1955 by Th. Förster, who proposed the concentration-quenching effect upon aggregation due to strong π–π stacking interactions in the organic dyes. Such effect is now commonly known as the ACQ (aggregation-caused quenching) effect [9]. In 1970, the ACQ effect was told as “common to most aromatic hydrocarbons and their derivatives” by J. B. Birks, in his book entitled *Photophysics of Aromatic Molecules* [10].

With the rise and development of new technologies, the twentieth century opened the doors to a new perspective about luminescent materials. In most modern applications, such as optoelectronic devices (as laser, optical storage, and OLEDs) and biomedical tools, fluorescence materials are used as solids and/or aggregates [11,12,13,14,15,16,17,18,19]. The need for materials suitable for such applications led to a growing interest in the design and synthesis of aggregate emitters.

The first pivotal report containing the concept of aggregation-induced emission materials is due to Tang and coworkers [20]. Aggregation-induced emission (AIE) compounds display a photophysical phenomenon in which molecular aggregates exhibit stronger emission than single molecules. In the following articles, the research group presented a silole derivative (hexaphenylsilole) non-emissive in solutions up to 80% water fraction. Above this threshold, strong fluorescence was observed due to the strong aggregation of the molecules. Contrary to the traditional ACQ effect, this effect was coined as “AIE”, and the molecules undergoing the AIE effect were termed “AIEgens” [21,22].

The unique AIE behaviour changed researchers’ way of thinking. The AIE operating mechanism is now under examination from a new applicative perspective due to the growing demand for advanced luminescent materials [23,24,25]. Milestones for AIE theoretical development are several scientific publications from 2001 up to now. First, the origins of the phenomenon were investigated, and several mechanisms proposed for an explanation of the AIE effect. In 2003, again Tang and coworkers proposed the restriction of intramolecular rotations (RIR) mechanism based on the study of hexaphenylsilole [26] and proved it as applicable to most AIEgens. When the single AIE molecule is dissolved in solution, the dynamic intramolecular rotations cause a fluorescence quenching by dissipating the exciton energy. Contrarily, upon aggregation, the restriction of the intramolecular motions suppresses the radiationless decay pathway. Despite this, the RIR process cannot explain some experimental evidence, and other effects were added in the mechanistic study of the phenomenon. The intramolecular vibrations of flexible moieties have been considered [27], generating an additional model named the “intramolecular vibrations (RIV) effect”. A generalised mechanism for AIEgens named “RIM” (restriction of intramolecular motions) was introduced in 2014 by combining RIR and RIV effects, so including rotation, vibration, bending, flapping, twisting, and other intramolecular motions concurring to the radiationless decay pathways [28,29,30,31,32]. A theoretical confirmation of the RIM mechanism was achieved by time-dependent density-functional theory [25].

Additional mechanistic models and theories were proposed to investigate the photo-induced process occurring in different AIE systems. Specifically, to predict AIEgens behaviour, other energetic parameters are involved and must be considered. The Duschinsky rotation energy [33,34,35,36,37,38] (due to the difference between the ground state and excited-state potential energy surfaces, calculated by the harmonic oscillator model); the reorganisation energy [39] (required to relax the structure and environment upon electron transfer); the formation of J-aggregates (causing a bathochromic shift in the absorption due to π–π stacking of the aromatic moieties) [22]. Finally, the restricted access to a conical intersection (RACI) model to analyse the global potential energy surface topology was introduced in 2013 by Blancafort and coworkers [38]. Conical intersections were described as regions of the potential energy surface where the ground and excited states are degenerate, and the probability of non-radiative internal conversion is maximal. RACI model generally leads to a forecast on the fluorescence yield (PLQY, Φ). This fundamental parameter is defined as the ratio of the number of photons emitted to the number of photons absorbed and derives from evaluating both the radiative and the non-radiative channels activated in the AIE molecule [40,41,42,43,44].

To conclude, a correct theoretical setting of the chemical system is crucial for the actual prediction of the photoluminescence yield of the AIE fluorescent dye. The observation of the effects involved in the activation of emission in the aggregate state gave scientists new ideas about AIEgens design.

### 1.2. AIEgens as the Novel Scientific Frontier for Cutting-Edge Technologies

The great potential of AIEgens is documented by the number of publications and citations about the topic, exponentially increasing since the first AIE report in 2001. To date, more than 2000 papers (articles and many reviews) have been published under the keyword “AIEgen”, and AIE topic was ranked no. 2 and no. 3 in the list of “Top 100 Research Fronts in Chemistry and Materials Science” by Thomson Reuters, respectively, in 2015 and 2013. In 2016, Nature Journal placed AIE-based dots between the four key materials for the novel lightening nanotechnology [45]. In the last 20 years, AIEgens have been deeply explored and their unique advantages over conventional ACQ molecules fully recognised. The implementation in AIEgens study is moved by the cutting-edge technologies demand, ranging from optoelectronic to sensing and bioimaging [46].

Many optoelectronic techniques greedily require emissive solid layers. The earliest application of AIEgen in optoelectronic devices was reported in 2001 as blue-emissive AIE siloles with excellent external quantum efficiency [47]. Several AIEgens have been designed with the precise aim of obtaining solid-state emissions. The nature of AIEgens matches the structural tunability and manipulability required to produce an optical device. In addition, the whole emission colour display, up to the NIR region, can be easily obtained by structural modifications in the AIEgen skeleton. 

Due to their versatility, AIEgens can be easily obtained in a polymeric- or macro-structured form by reaction of functionalised fluorophores or by doping the fluorophores in a polymeric matrix. Macromolecular materials are ideal candidates for optoelectronic applications based on solid-state layers, such as organic light-emitting diodes (OLEDs, optical waveguides, and luminescent solar concentrators, or even soft-matter-based devices such as light-emitting electrochemical cells (LECs) and liquid-crystal displays [48,49,50,51,52]. 

Other significant applications of AIEgens are fluorescence chemosensors and multiple-stimuli (such as pH, electromagnetic radiation, morphology) responsive materials [53,54,55,56]. AIEgens can be utilised as fluorescent indicators/markers to characterise supramolecular interactions and macromolecular motions in the solid state, in an aggregate gel phase or even in a water-concentrated solution. In the presence of the target analyte, the AIE channel can be activated or deactivated, resulting in an optical recordable signal. In 2005, Tang and coworkers produced chemosensors based on silole as sensors for the regioisomers of nitroanilines [57] and in 2010, Park and coworkers promoted AIEgens as stimuli–responsive materials. To date, several AIE-based chemosensors were reported for the detection of ions, metals, small organic molecules, and biological targets [58,59,60,61,62,63,64,65,66].

Porous crystalline solids such as inorganic nanoparticles (NPs), metal–organic frameworks (MOFs), covalent organic frameworks (COFs), and carbon nanotubes (CNTs) are materials where the choice of suitable AIE active fragments can produce highly engineered composite materials with unique properties [59,60,61]. In the composite material, the AIE moieties interact with a regular structure resulting in tunable electronic and optical properties. Low self-quenching and a highly modulable inner chemical environment can be achieved. Thanks to the porous architecture, such nanostructured AIEgens are promising candidates for fluorescence sensing with high sensitivity and selectivity toward specific analytes [61,62,63,64,65,66]. 

Finally, photoluminescent devices for biomedical uses take advantage of the fluorescence characteristics of AIEgens [63]. The incorporation of AIEgens in bio-compatible materials through various methods (covalent or coordinate bonds, noncovalent interactions) provides AIEgen-based composite biomaterials for several uses. Bio-engineering of nano-sized probes based on AIEgens produced highly specific and responsive systems. In 2012, Tang and Liu and coworkers developed AIEgen bioprobes for cell-imaging and monitoring of biological processes [64]. Biocompatible AIEgen-based NPs were designed still by Tang for biological and biomedical applications [65]. Due to their permeability and retention, biocompatible AIE-based dots and NPs were also applied for in vivo cancer imaging and diagnostics [65,66]. Again, Tang and coworkers, in 2018, reported a simple approach to link AIEgens to bio-relevant species for biological labelling and monitoring [67]. 

Lessons learned from these advanced tools provide new ways of thinking about the most relevant in vivo applications. 

### 1.3. Metal-Containing AIEgens and The Role of Zinc (II) Ion

In the following years, many purely organic, aromatic and heteroaromatic RIM undergoing AIEgens were described. Specifically, AIEgens bearing cores such as tetraphenylethene, thiophene-triphenylamine, tetraphenylpyrazine, quinoline, 9,10-distrylanthracene, dithiole, and derivatives have been extensively reviewed in recent articles [21,25,63,68,69,70,71,72,73,74].

Despite the obvious advantages of organic fluorophores, which are singlet emitters, heavy atoms such as transition metals display triplet emission. The related spin–orbit coupling leads to efficient singlet–triplet state mixing so that the presence of a heavy atom can improve the photophysical properties of π-conjugated ligands [75,76,77,78]. The formation of metal complexes represents a strategy for obtaining new luminescent materials with long luminescence lifetimes, large Stoke’s shifts, and high PLQYs in the visible region. Metal complexes are potentially unique luminophores with highly tunable structures and photophysics. Specifically, the d-block metal complexes are rich in different charge-transfer electronic states (MC, Metal Centered; ICT, Intramolecular Charge Transfer; ILCT, Intra-ligand Charge Transfer; LC, Ligand Centred; MLCT, Metal to Ligand Charge Transfer; LL’CT Ligand to Ligand Charge Transfer) and local π–π* transitions on the ligands [78,79,80,81,82,83,84].

The origin of the AIE effect in transition metal complexes can be explained based on the competing radiative and non-radiative de-excitation paths. The presence of the coordinated metal leads to a selective activation or blocking of these channels. Besides, blocking the non-radiative deactivation pathway, the presence of the coordinated metal can also produce a more efficient emitting state of the organic part in its aggregate state. In fact, the electronic charge in the complex can be transferred between different molecular moieties, involving electron transfer between metal cation and ligands. This transfer causes an alteration in the energy levels and in the emitting state. The AIE effect is potentially ascribable to simultaneous changes of the emitting state and activation of RIM and/or RACI mechanisms. Obviously, the activated AIE channel can be predicted only with an in-depth theoretical analysis [85,86].

Several transition metals such as Ir(III), Pt(II), Re(I), Ru(II), Os(IV), Au(I), and Pd(II) are reported to cause the activation of the AIE channel [78]. The development of AIE-active complexes seems to be a promising strategy for many AIE systems where the metal is strictly involved in the emission process. In all cases, ML and/or LM transfer must be expected in the emissive complexes.

Among the wide variety of metal-based luminophores, experience as researchers moves us to recognise a unique role of zinc (II) cation [87]. In the growing demand for high-performance devices, sustainability is a relevant parameter. Zinc, as a small eco-friendly cation, is a good alternative to other hazardous or expansive metals. Zinc complexes claim unique peculiarities which endorse the use in the production of AIE undergoing materials. Specifically, zinc (II) complexes exhibit fluorescence intensity and/or colour tuning in dependence on the electronic pattern of the ligands and the coordination pattern imposed by the ligands. In fact, in the d^10^ closed-shell zinc (II) ion, d–d electronic transitions are not expected. The lowest energy excited states are mainly LCT transitions (such as ICT and ILCT bands) and/or LL’CT transitions, rarely LMCT due to s or p empty orbitals of the metal [7].

**Scheme 1:**
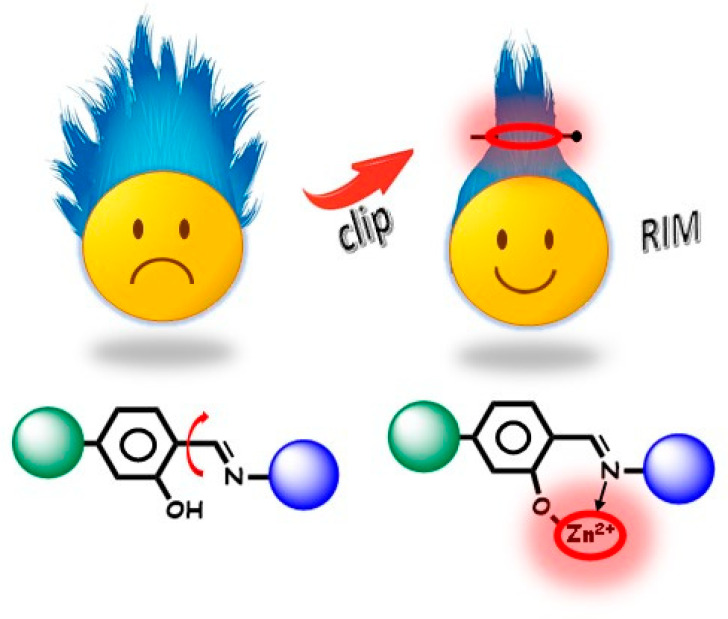
The “clip approach” in Zn AIEgens.

That is what we mean as a “clip approach” (Scheme 1). In most cases, the cation acts as a constraint for the ligand by locking it into a favourable emissive conformation, with few electronic effects mostly due to π–π* LCT transitions. Chelation enhanced fluorescence (CHEF mechanism) is often involved in zinc (II) binding as the result of the stabilisation of the excited state in poorly emissive ligands [14]. Therefore, the role of zinc (II) cation can be to turn a non-emissive ligand in an AIEgen molecule upon coordination. Alternatively, zinc (II) can tune and/or increase the emissive properties of the AIE ligand through suitable assembly of AIE portions. In addition to the “optically innocent clip role”, zinc cation offers the ability to give rise to the most varied architectures through coordination of organic ligands, self-assembly of two and three-dimensional macrostructures, aggregation of nanomaterials, and production of regular or amorphous structures [88,89,90,91,92,93,94].

Despite several studies encompassing the topic of AIEgens, in this review, we will focus our attention on the role of Zn(II) in the design of AIE active complexes and materials. Our main aim is to give a unified overview of AIEgens involving zinc (II) cation (abbreviated as Zn AIEgens). With the term “Zn AIEgens”, we mean: the zinc (II) complexes exhibiting AIE properties; the AIEgens giving a fluorescence response in the presence of zinc (II) cation; the zinc (II) complexes exploiting an AIE mechanism to detect specific analytes. In short, with this review, we wanted to answer the question: what zinc (II) is dealing with AIEgens. 

Considering the novelty of the topic, we cut the last 5 years (years 2016–2021) as representative of the recent scientific production. Selected examples were reported to elucidate the state-of-the-art, starting from the simple phenomenological approach and considering different typological and chemical units and structures. We will focus on the recent Zn AIEgens based on the synthetic novelty, research completeness, fluorescence response, theoretical deepening, and relevant applications. Specifically, we reviewed the literature and organised the discussion in the following sections:

1. Newly developed Zn AIEgens;
2. Zn AIEgens for OLEDs and other optical technologies;
3. The role of zinc (II) in AIEgen chemosensing;
4. Zn AIEgens for cell imaging.

Scientific literature, including reviews, were consulted to understand the structure, the optical response, and the operating mode of Zn AIEgens. Finally, we underlined the representative applications for the targeted biological and medical scope. By use of a quick glance representation which should be considered an integral part of the discussion, we propose an intuitive overview of groups of structures examined according to the application area.

## 2. Newly Developed Zn AIEgens

The examples reported in this section can be considered the first level in the study and development of novel targeted Zn AIEgens. The starting point is represented by the design and synthesis of simple small organic molecules, even non-emissive, acting toward Zn(II) as ligands or chelators. In the formation of the complex, the organic units are subjected to constraints (RIM), and therefore, the final complex undergoes the AIE effect. The zinc complexes are expected to meet specific requirements, such as high PLQYs in the aggregate phase, photostability, large lifetime, and Stokes shifts. The study of the solvent effects is relevant. An easy test to assess the AIE nature of the compound consists of recording emission intensity as the ratio of non-solvent/solvent increases [95]. Emission is expected to grow as the aggregation increases. Many solid-state emitters are *de facto* AIEgens. A complete analysis of the solid-state emission properties achieved by the AIE mechanism and the relationship with the structural data is a central point to these articles. Relevant information is inferred by a supporting theoretical analysis of the energetic transitions involved in the AIE response [28,69,96,97,98,99].

The synthetic Zn AIEgens reported in this section are referred to as “potentially useful” materials for optoelectronic applications and/or optical techniques. On the other hand, articles including targeted applications will be reported in Section 3.

**Figure 1:**
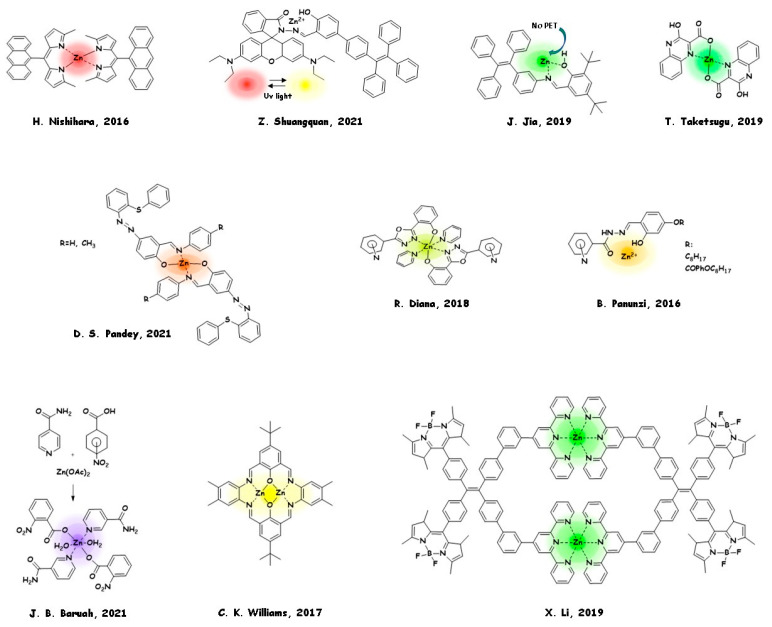
Zn AIEgens obtained by encumbered nitrogen and/or oxygen-donor-based ligands.

**Figure 2:**
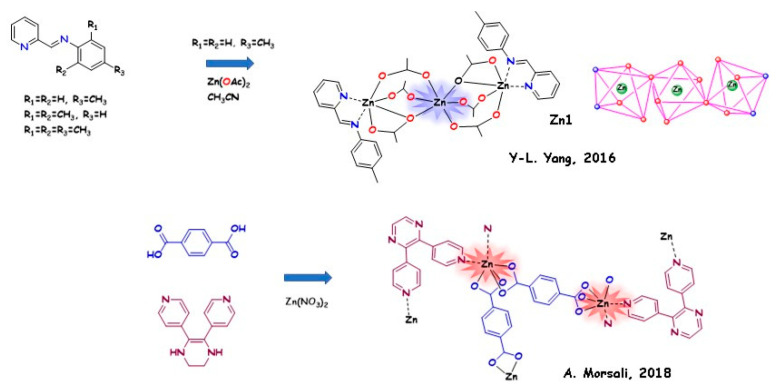
MOFs acting as highly fluorescent Zn AIEgens.

In Section 2.1, we will report the most relevant examples of novel Zn AIEgens obtained by encumbered nitrogen and/or oxygen donor-based low-molecular-weight ligands (RIM effect undergoing complexes). Our discussion will be articulated based on structural similarities and differences and on the AIE activated channel. The related structures are summarised in Figure 1, accordingly with discussion. In Section 2.2, we referred to a few relevant examples of metal–organic frameworks acting as highly fluorescent Zn AIEgens. The related structures are reported in Figure 2.

### 2.1. RIM Designed Zn AIEgens

The most common and easy approach to obtain Zn AIEgens is to enhance steric hindrance by adding bulky substituents to the chelating ligands. The ligand undergoes a conformational block upon coordination, producing the RIM fragment. A selection of relevant examples of synthetic Zn AIEgens undergoing the RIM effect is grouped based on chemical/structural features. As it will be discussed, some peculiar skeletons able to cause the RIM effect are recurrent.

In 2016, Hiroshi Nishihara [100] and coworkers studied the solid-state PL emission of seven encumbered tetradentate bis(dipyrrinato)zinc (II) complexes. The bulky effect [101,102,103] due to the meso-aryl group in the dipyrrin ligands proved to affect the emissive properties of the dipyrrin Zn(II) complexes. The conformation of the meso-aryl group in the excited state greatly affects the nonradiative decay pathways. In solution, PLQYs increased as the solution turned into aggregate state PLQYs by adding a non-solvent. In the solid-state, each complex showed different emission wavelengths and spectra depending on the crystal packing structure. With respect to the solution behaviour, the different PLQYs and lifetimes recorded on the crystalline complexes proved the activation of different photophysics in the solid state. A *O*,*N*,*O* hydrazone tridentate ligand was synthesised by Zang Shuangquan and coworkers [104] in 2020, starting from Rhodamine B 2-Hydroxy-5-tetraphenylethene-benzaldehyde hydrazone. A typical AIE moiety, tetraphenylethene (TPE) group, was introduced as a bulky substituent, able to undergo fluorescence resonance energy transfer (FRET) [105] process from TPE moiety to rhodamine B moiety. As a result, the complex obtained by reacting the composed ligand with zinc (II) cation is a Zn AIEgen and exhibits reversible fluorescence colour and intensity changes (red/yellow) upon UV light photo-induced irradiation.

Common *salen* type ligands with encumbered substituents are excellent candidates in building Zn AIEgens, zinc cation acting as a constraint of the structure. In some cases, intriguing fluorescence behaviour can be achieved by specific substituents onto the simple Schiff-base *O,N* moiety. In 2019 Junhui Jia and coworkers [106] synthesised a tetraphenylethylene-functioned Schiff-base ligand (TPEMO) with multifunctional behaviour. The molecule itself is an AIEgen and shows reversible mechano-fluorochromism after grinding. It was found responsive towards Zn^2+^ (LOD = 8.05 × 10^−8^ M) and CO_3_^2−^ ions. The sensing mechanism of the molecule with Zn^2+^ was ascribed to the inhibition of PET transfer and consequently of the ESIPT mechanism. Very recently, Daya Shankar Pandey and coworkers [107] studied two novel azo-based Schiff-base functionalised ligands. Again, the ligands themselves are AIEgens, potentially useful as solid-state fluorescent sensors for acidic vapours. Upon irradiation with UV light, the N=N azo bridge exhibits *cis–trans* isomerisation both in the ligands and in their zinc (II) complexes. Comparative photoswitching studies and DFT analysis was performed on ligands and complexes based on X-ray analysis. In 2019, Tetsuya Taketsugu and coworkers [108] performed a complete theoretical study on a Zn AIEgen derived from 3-hydroxy-2-quinoxalinecarboxylate showing ESIPT emission in the solid state, even though the ligand does not undergo ESIPT emission. TD-DFT analysis was employed to explore the role of the zinc atom in the emission mechanism, resulting in a structural change of the ligand from the lactam form (3,4-dihydro-3-oxo-2-quinoxalinecarboxylic acid) to the enol form (3-hydroxy-2-quinoxalinecarboxylic acid) in the presence of zinc (II). Emission was imputed to LC transitions of the ligand.

A systematic study of isomeric Schiff-base zinc complexes showing relevant differences in their crystalline pattern and emission behaviour was published in 2018 by Rosita Diana and coworkers [109]. Three bidentate *N*,*O* Schiff-base ligands contained a 2,5-disubstituted-oxadiazole unit and an *ortho/meta/para*-substituted pyridine unit. The reactions of the ligands with zinc (II) acetate in pyridine gave crystalline complexes with the general formula Zn(L)_2_py_2_. In the solid phase, PLQYs (ranging from 16 to 75%) and emission colour (in the red–green–blue or RGB shape) were recorded in dependence on the structural pattern. The reaction with zinc cation self-assemblies the *meta* derivative ligand in a stable glassy network, with Zn(L)_2_ general stoichiometry and PLQY = 44%. The same research group (2016, Barbara Panunzi and coworkers [110]) produced aroyl- and acylhydrazones ligands acting as differently substituted *O*,*N*,*O* tridentate pincers to zinc. The photophysical properties of the tridentate complexes in the crystalline phase were explored. Additionally, in this case, the one-dimensional crystalline coordination polymer obtained from the *meta* derivatives showed high PLQY (74%) in the solid state. 

The results achieved in both articles suggested that the polymeric or reticulated macrostructures obtained by zinc-driven self-assembly provide emission enhancement. Both in the case of an amorphous and crystalline material, the tight structure with optically “innocent” zinc nodes could guarantee a more efficient electron hopping in the whole macrostructure.

The role of the metal in assembly together non-fluorescent components was examined in 2020 by Jubaraj B. Baruah and coworkers [111]. By zinc-assembly reaction of nitrobenzoate and pyridine-3-(or-4)carboxamide, five different complexes were obtained. The role of MLCT in the resulting Zn AIEgens was explored by DFT calculations. The frontier molecular energy levels of different combinations of the positional isomeric complexes were ascertained. In such cases, the d^10^ cation resulted in contributing to the emission through CT transitions. The highest PLQY (13.56%) was recorded for the non-ionic complex (di-aqua)bis(pyridine-3-carboxamide)di(2-nitrobenzoato)zinc (II) monohydrate at 439 nm. The AIE effect caused by adding water to a DMSO solution was studied. As expected, the participation of water molecules to form aggregates with the complexes caused increased emission intensity up to a characteristic limit. Above that concentration, emission quenching was recorded due to the equilibrium between the complex and the hydrate cation.

Other structures causing the AIE effect by steric constrain are metallo–supramolecular architectures, achieved by zinc-driven self-assembling reaction. Specifically, emissive or non-emissive polyfunctional ligands can be assembly in macrocycles or metallocycles by reaction with zinc cation. The final metal complex undergoes restrictions of intramolecular motion increased with respect to the starting organic moieties and are expected to be a Zn AIEgen. Charlotte K. Williams and coworkers in 2017 [112] investigate the solid-state light emission of seven zinc *salphen* (*salphen* = *N*,*N*′-bis(salicylidene)-1,2-phenylenediamine) complexes in a macrocycle pattern. By gradual addition of a non-solvent to the complexes dissolved in chloroform, a visible aggregation was induced, and emission enhancement was recorded. The complexes showed yellow to orange–red emission in the solid state (PLQE = 1–5%), displaying up to a 75-fold increase in peak emission intensity upon aggregation, in the best case.

Terpyridine (TPY) is a tridentate ligand with three coordination sites belonging to *N*-heteroaromatic rings. Owing to the strong chelating ability, TPY derivatives can form stable complexes with several different transition metal ions. Xiaopeng Li and coworkers in 2019 [113] studied self-assembly of emissive metallocycles with tetraphenylethylene (TPE), boron-dipyrromethene (BODIPY), and terpyridine (TPY). Combining the three blocks into one system by zinc coordination driven self-assembly reaction, a metallocycle dimer with typical AIE characteristics was produced. Interestingly, by combining the three fluorophores into a metallo–supramolecular architecture, emissive properties were recorded both in solution and in the aggregated state.

### 2.2. AIE Zn-MOFs

Metal-organic frameworks (MOFs) are hybrid organic–inorganic crystalline materials derived by the regular array of metal cations (or clusters) surrounded by organic linkers. The metal ions act as nodes binding the organic fragments into a regular cage-like structure. They are a subclass of self-assembly coordination polymers (CPs) with a tridimensional feature. Due to this hollow structure, MOFs have a porous internal surface area. The small-sized internal cavity of the materials is nanoscaled (in at least one dimension, between 1 and 100 nm). Therefore, MOF research adopts a scientific approach to nanotechnology. Professor Omar Yaghi at UC Berkeley in the late 1990s (“Design and synthesis of an exceptionally stable and highly porous metal-organic framework”) presented MOFs for the first time, and they rapidly become a cutting-edge research field. The synergistic effects of structures and compositions make MOFs fascinating examples of unique structural design and tunable properties. Different metal atoms and organic linkers lead to MOFs selectively absorbing targeted molecules /ions. Tailored MOFs offer great potential due to their optical, electronic, opto-electronic, or sensing properties [114,115,116].

In recent decades, MOFs with luminescence characteristics (luminescent MOFs, LMOFs) has been considered as the new pathway for the fabrication of solid-state luminescent nanomaterials. AIEgen ligands can be employed as building blocks in the MOFs construction. To retain the fluorescence of AIE linkers or activate the AIE channel, the closed-shell zinc (II) ion with low-lying d-orbital energy can be preferred to classic heavy metals. Zn(II) nodes afford MOFs with strong luminescence due to constraining effect on the AIE ligands.

In this section, we reported a few relevant examples of LMOFs containing zinc (II) and acting as highly active AIEgens (we will use the term “AIE Zn-MOFs”). In Figure 2, we reported a schematic representation focusing on the structural feature.

The Schiff-base pattern also demonstrated to be a winner strategy in building LMOFs. In 2016, Yu-Lin Yang and coworkers [117] used five Schiff-base ligands containing a *N*-(pyridine-2-yl) fragment with different alkyl substitutions on the phenyl ring for the synthesis of Zn(II)/Cd(II) complexes. The structures of the ligands can direct the formation of 3D supramolecular LMOFs due to hydrogen bonds and π–π interactions. Nine Zn(II)/Cd(II) complexes were obtained, displaying deep blue emissions (401−436 nm) in acetonitrile solution and light blue/bluish green emissions (485−575 nm) in the solid state. Structural analysis gave information on the crystalline packing of the complexes. PL measurements recorded in CH_3_CN/H_2_O mixtures gave evidence of an effective AIE behaviour for the Zn1 (in Figure 2) aggregate complex.

Ali Morsali and coworkers [118] in 2018 synthesised a turn-on AIE-based MOF by in situ ligand fabrication and employing different valence-shell cations, with the aim of investigating the effect of metal nodes. The coordination of 5,6-di(pyridin-4-yl)-1,2,3,4-tetrahydropyrazine (AIE fluorophore) and terephthalic acid ligand with Zn(II), Co(II), or Cd(II) leads to the preparation of strongly luminescent MOFs (named “TMU-40(Zn)”, “TMU-40(Cd)”, and “TMU-40(Co)”). The formation of rigid frameworks improved the fluorescence of the organic parts more with zinc than with the other two metals (PLQY = 38.2% with respect to 31.17% and 11.69%, respectively).

## 3. Zn AIEgens for OLEDs and Other Optical Technologies

The basic research on RIM undergoing Zn AIEgens was a platform to produce effective technological devices. Solid-state emitters have a great potential for the development of novel optoelectronic technologies, such as OLEDs and other optical tools. The growing demand for low-cost light-emitting devices focused attention on the abundant, nontoxic, versatile zinc cation. Zinc (II) complexes are a potential alternative to expensive transition metals complexes of iridium, osmium, and platinum, and can represent excellent luminescent and electron-accepting/transporting materials. They can be employed as emitter layers in a variety of forms: solid powders, dyes dispersed in host solid and/or gel matrixes, and metallo–polymers. Since the first OLED based on a Schiff-base zinc (II) complex developed by Hamada in 1993 [119], the research on highly efficient emissive zinc (II) complexes continued. Zinc complexes also have a potential for the construction of white OLEDs (WOLEDs) due to the tunable emission colour. To date, reports on effective zinc (II) complex-based OLED are rare, and there is no commercialisation. Nonetheless, the study of Zn AIE systems makes room for new ideas, and Zn AIEgens claim a role in the novel technological frontier.

**Figure 3:**
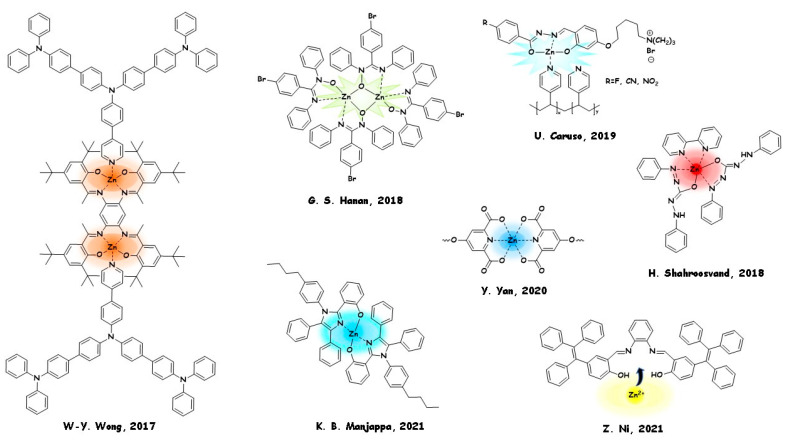
Zn AIEgens for optical applications.

In this section, we will discuss the most relevant examples of opto-applications of Zn AIEgens, from OLEDs and LECs to other cutting-edge technologies. In Figure 3, we collected the related structures, referring to the most PL active architectures.

Wai-Yeung Wong and coworkers in 2017 [120] presented four novel *salphen* complexes bearing pyridyl functionalised ligands with dendritic structures. OLED devices based on the novel zinc complexes were fabricated by a solution process. The electroluminescence (EL) properties achieved by zinc (II) complexes were checked: peak luminance (L_max_) of 3589 cd m^−2^, maximal external quantum efficiency (η_ext_) of 1.46%, maximum current efficiency (η_L_) of 4.1 cd A^−1^, and maximal power efficiency (η_P_) of 3.8 lm W^−1^. Garry S. Hanan and coworkers in 2018 [121] synthesised a dinuclear complex of zinc (II) with 4-bromo-*N*,*N*′-diphenylbenzamidinate N-oxide. This compound is a Zn AIEgen and was used as a dopant in a co-host matrix of the emissive layer used in the fabrication of a solution-processed white–green WOLED. A luminance efficiency and power efficiency of 1.12 cd/A and 0.30 lm/W, respectively, were obtained.

RGB (red, green, blue) metallopolymer emitters with potential in WOLEDs preparation were examined by Ugo Caruso and coworkers in 2019 [70]. Three aryl-hydrazone *O*,*N*,*O* tridentate ligands with a different electron-withdrawing substituent and a charged moiety were prepared by grafting ligand-Zn(II) coordination fragments onto commercial poly-(4-vinylpyridine). Three RGB emissive polymers were obtained. By grafting a suitable mix of the three different coordination moieties, an efficient single-component white-light blue emissive metallopolymer (CIE: 0.30, 0.31) was prepared. The same material resulted emissive both in the solid phase and mixed with an ionic liquid, so resulting employable in WOLEDs and in LECs preparation. A rare example of LEC was prepared by Hashem Shahroosvand and coworkers in 2018 [122], based on a blend of the cationic complex [Ru(bpy)_3_]^2+^ and a neutral zinc (II) complex derived from diphenylcarbazone ligands. The crystal structure of the Zn fluorescent complex was examined, and the assignment of ground- and excited-state transitions was achieved by TD-DFT analysis. The deep red LEC shows a brightness (740 cd m^−2^) and luminous efficiency of 0.39 cd/A at a low turn-on voltage of 2.5 V.

Recently, Kiran B. Manjappa and coworkers [123] performed a comprehensive structural, spectroscopic, and theoretical analysis of a bisimidazolyl zinc AIEgen complex. This complex emits cyan fluorescence with a high quantum efficiency (83%), leading to two different applications. First, it was used for the fabrication of an OLED, with a maximum brightness of 15,000 cd/m^2^ (16 V), and external quantum efficiency close to 3.8% (comparable with state-of-art of the purely fluorescent non-thermally activated OLEDs). Moreover, the complex was employed in the fabrication of a latent fingerprint (LFPs) investigation tool. Fingerprint detection is a crucial area in forensic science and is based on fluorescent dyes. Under UV light, the finger marks on the surfaces can be photographed by a digital camera resulting in more clearly visible and examinable fingerprints. Dispersed in poly(methyl methacrylate), the dye can also be used as a straightforward security ink for anticounterfeiting applications. Moved by a similar approach, Yun Yan and coworkers in 2020 [124] reported a water-based polyion anticounterfeiting micellar ink displaying full-spectral RGB emission colours by a combination of AIEgens and luminescence of rare earth metals. Blue emission was achieved from the AIE fluorophore tetraphenylethylene (TPE) linked by zinc cation into coordination supramolecular polymers. Recently, Zhonghai Ni [125] developed an ESIPT-based AIEgen Schiff-base compound containing a tetraphenylethene skeleton preventing the ACQ effect. Both the absorption and fluorescence spectra are water- and Zn(II)-responsive. The multifunctional ligand sensor was successfully applied as an inkless rewritable paper and as a colourimetric/fluorescent dual-channel sensor for zinc (II) ion.

## 4. The Role of Zinc (II) in AIEgen Chemosensing

Some of the established methods for trace elements or molecules detection require sophisticated spectrometry and spectroscopy as accurate and sensitive-but-expansive analytical techniques. Today, many sought-after substitutes are low-cost luminescent probes for on-site determinations.

Fluorescence sensors for zinc detection are a relevant research area. Zinc (II) ion plays a crucial role in many chemical, biological, and environmental processes. From a physiological point of view, zinc (II) cation is an essential metal ion for many biological processes, such as the formation of zinc finger proteins, the neural signal transmission/modulation [9,10], the regulation of gene expression [11], and various catalytic activity [8]. The mechanism of sensing fluorescence turn-on of most zinc (II) chemosensors is based on MLCT, CHEF effect, PET, and C=N isomerisation. Up to now, many classic organic fluorophores (such as coumarin, fluorescein, rhodamine, and cyanine) used for sensing applications undergo ACQ effect at high concentrations or in the aggregated state. Contrarily, organic AIE receptors can be advantageously employed in sensing zinc (II). According to a common strategy, when an organic AIEgen is dissolved in solution, a turn-off of fluorescence is expected. The addition of zinc can induce a turn-on of the fluorescence by different mechanisms: the restriction of intramolecular motions (chelation-aided rigidification, CAR); the decrease of solubility of the sensor, which causes the formation of emissive aggregates (cleavage-triggered aggregation, CTA); the metal-caused blocking of nonradiative pathways (metal-bridged crosslinking, MBC); the metal-caused blocking of intramolecular motion (coordination-induced complexation; CIC) [126]. Therefore, the sensing mechanism involves the active role of the metal in the PL turn-on of the solution. A linear relationship between PL increase and zinc concentration can be exploited to achieve quantitative information.

On the other hand, integrates Zn-containing chemosensors claim a role in the wide field of fluorescence sensing probes. Probes based upon the AIE strategy proved to be attractive and versatile tools for sensing biologically relevant small molecules or ions. AIE chemosensors acting in water solution shows potential in imaging techniques due to their high sensitivity, fast response, low cost, and technical smartness. Fluorescent probes exhibiting AIE behaviour thanks to the presence of zinc (II) cation in a structural key position are an interesting analytical application of Zn AIEgens. The role of the zinc (II) ion is crucial in the turn-on (or turn-off) of the sensor emission. Undergoing coordination-decoordination equilibria, zinc (II) dissolve and reform AIE-active compounds depending on the presence of the analyte.

In this chapter, we will refer to the most relevant examples of AIEgens sensors involving zinc (II) cation as an analyte or as a part of the sensor. Specifically, our discussion will be articulated based on the target analyte as follows:

- AIE sensors designed for selective detection of zinc (II) ion (Section 4.1)
- Zinc (II) containing AIE systems able to detect several target analytes (Section 4.2 and Section 4.3).

We will explore and discuss single-analyte or multiple-targeted sensors on the basis of the structural pattern and the sensing mechanism.

### 4.1. AIEgens for Zinc (II) Sensing

**Figure 4:**
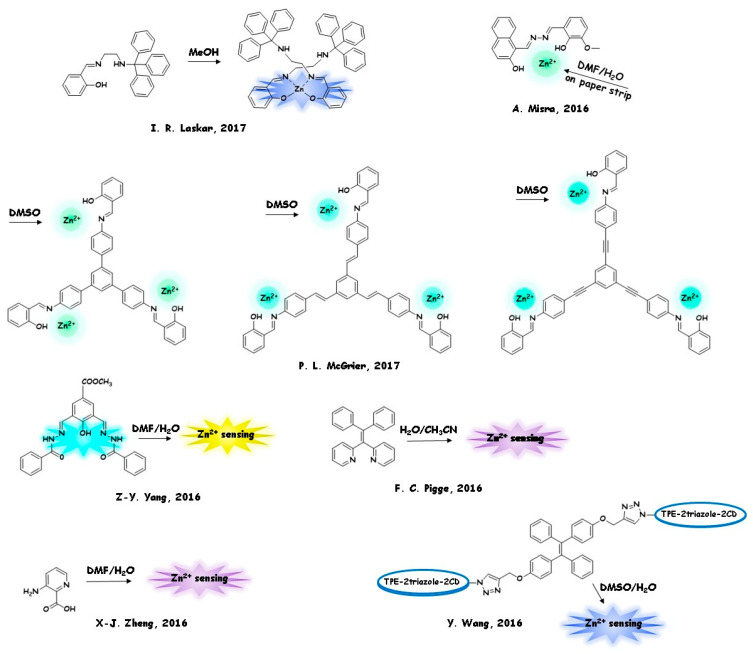
AIEgens acting as zinc (II) mono-channel sensor.

**Figure 5:**
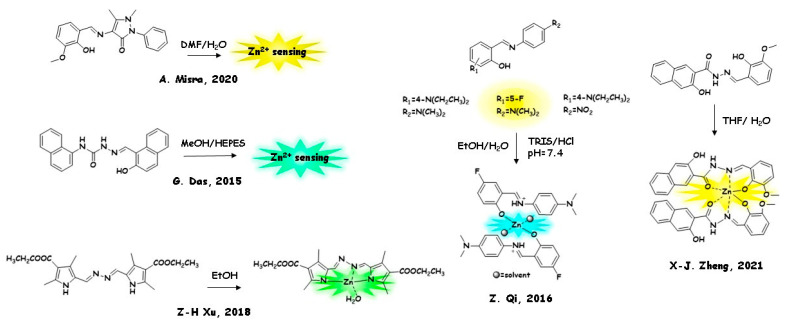
AIEgens acting as zinc (II) multi-channel sensor.

**Figure 6:**
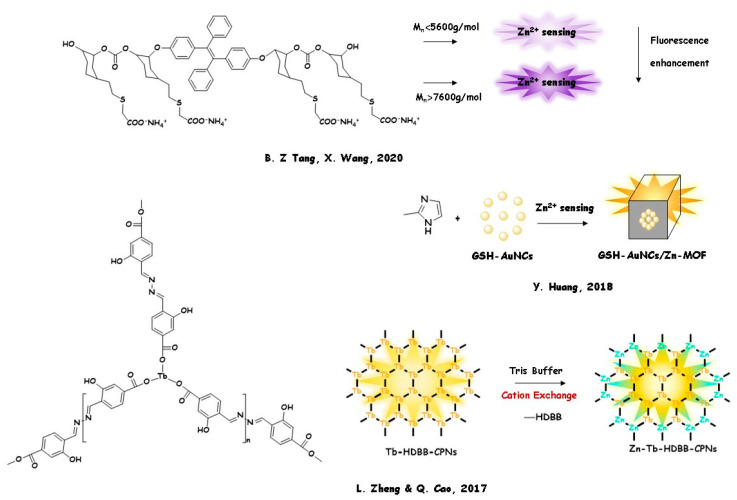
Macro-structured AIEgens acting as zinc (II) sensor.

In this section, we will refer to a selection of relevant AIEgens acting as mono-channel zinc sensors (Figure 4), as multi-channel zinc sensors (Figure 5), and macro-structured AIEgens acting as zinc (II) sensors (Figure 6).

Schiff-base ligands are good candidates in building zinc sensitive AIEgens. Inamur Rahaman Laskar and coworkers in 2017 [127] synthesised an RIR undergoing chelate, with J-aggregate in the crystalline phase. The sensor [2-(2-(tritylamino)ethylideneamino) methyl phenol] exhibits irreversible mechanoluminescence with an emission colour change from blue to green upon grinding of the powder sample. The ligand proved to be a selective sensor showing PL turn-on (23 folds the unbonded sensor) with LOD = 0.064 ppm upon zinc (II) addition. Three analogue AIE Schiff-base ligands containing 1,3,5-triarylbenzene, -tristyrylbenzene, and -tris(arylethynyl)-benzene cores were published in 2016 by Psaras L. McGrier and coworkers [128]. Their AIE behaviour was attributed to an ESIPT process, and the ligands are turn-on responsive to zinc (II) and copper (II) cations in DMSO solution. Ajay Misra and coworkers in 2016 [129] proposed an AIE fluorescence probe, 1-(2-hydroxynaphthylmethylene)-2-(3-methoxy-2-hydroxybenzylidene) hydrazine, which resulted in being highly selective toward zinc (II) based on a CHEF/AIE feature. Fluorescence turn-on was detected in DMF/H_2_O. Zheng-yin Yang and coworkers in 2016 [130] designed a bis Schiff-base AIEgen which detected Zn^2+^ ions in a ratiometric way (a ratiometric fluorescence sensor measures emission intensities at two or more wavelengths to accurately detect changes to the local environment/analyte), due to an ESIPT process.

In addition to classical Schiff-base type ligands, differently structured AIE chemosensors were successfully employed, with a fluorescence turn-on performance toward zinc (II). F. Christopher Pigge and coworker in 2016 [131] proposed three AIEgens, 2,2′-bis(pyridyl) tetraarylethylenes type ligands, for their ability to selectively detect zinc (II) ions in aqueous solution. In the same year, Xiang-Jun Zheng and coworkers [132] produced a 3-aminopyridine-2-carboxylic acid ligand that is AIE-active and zinc (II) responsive. RIR and RIV effects were activated by hydrogen bonds, resulting in rigidity enhancement of the molecule. Still, in 2016, Yong Wang and coworkers [133] produced a tetra-phenyl ethylene triazole-bridged sensor (TPE-2triazole-2CD). The sensor contains a cyclodextrin moiety which has a unique hydrophobic cavity able to encapsulate different guest molecules and a hydrophilic surface due to the hydroxyl moieties on the rings. The elaborate sensor shows a highly selective turn-on florescence response towards Zn^2+^ in neutral environments and potential biocompatibility.

AIE-based multi-responsive zinc (II) probes (Figure 5) are a growing research field. Multianalyte AIE sensors, or generally multifunctional AIE sensors, are highly desirable tools for many applications [134,135,136]. Ajay Misra and coworkers in 2020 [137] produced an antipyrine-based fluorescent probe, 4-[(2-hydroxy-3-methoxy-benzylidene)-amino]-1,5-dimethyl-2-phenyl-1,2-dihydro-pyrazol-3-one exhibiting turn-on selective fluorescence sensing toward Al^3+^ and Zn^2+^. In 2016 Zhengjian Qi and coworkers [138] synthesised three Schiff-base derivatives with electron acceptor and donor substituents exhibiting AIE properties due to an ESIPT process. Their response toward both zinc (II) cation and pH parameters was assessed, and the proposed binding mode at pH = 7.4 is illustrated in Figure 5. Another ESIPT-based turn-on AIE sensor was recently synthesised by Xiang-Jun Zheng and coworkers [139]. N-(3-methoxy-2-hydroxybenzylidene)-3-hydroxy-2-naphthahydrazone ligand demonstrated its ability to differently coordinate Zn^2+^ and Cd^2+^ ions. The sensor selectively recognises the metals due to different emission peaks (at 560 and 645 nm, respectively in solution). Interestingly, the ions were recognised quantitatively in water, and qualitatively in the solid state via test paper (light yellow emission).

A more elaborate multianalyte performance was realised in 2015 by Gopal Das and coworkers [140]. They synthesised an AIE probe with both an anion and a cation binding site. The ligand resulted in being turn-on fluorescence responsive and selective toward Al^3+^ and Zn^2+^. It can also detect Cu^2+^ in mixed buffer medium, and F^−^ in acetonitrile through colorimetric response. Zhi-Hong Xu and coworkers in 2018 [136] proposed an AIE active bis-hydrazone selectively detecting Cu^2+^, Zn^2+^, and Hg^2+^ by monitoring absorption (for copper ion) and fluorescence (for zinc and mercury ions) spectral patterns.

Examples of polymeric AIEgen sensors for zinc (II) are rare and intriguing due to the relationship between structural pattern and AIE response (Figure 6). Ben Zhong Tang and Xianhong Wang and coworkers [141], in 2020, synthesised conjugated polyelectrolytes with adjustable molecular weight (M_n_) through a combination of controllable polymerisation and aggregation-induced emission (AIE) techniques. The AIE-active polyelectrolytes are responsive to Zn^2+^ ions with a fluorescence turn-on response associated with M_n_. Specifically, in the polymers with M_n_ < 5600 gmol^−1^, the aggregation of the nanoparticles is due to the diffusion of the cation into the interstitial space between the COO− groups. On the other hand, the long-wrapped chains of polymers with M_n_ > 7600 gmol^−1^ hinder the diffusion of Zn^2+^, which in turn can coordinate between different nanoparticles, enlarging the size of the aggregates. As expected, the fluorescence increases much more in the second case. The observed phenomena provide a relationship between AIE behaviour and structural parameters, assuming a zinc ion detection of nanoparticle sizes.

Coordination polymers obtained by self-assembly of binding organic ligands with metal cations can be obtained in the nanoscale. CPs derived by nanoparticles (CPNs, sized between 1 and 100 nm) can be formed by self-assembly of a metal ion and organic ligands and provide a unique platform for designing nanosized AIEgen. A relevant example of zinc (II)-responsive CPN was reported in 2017 by Liyan Zheng and Qiue Cao and coworkers [142]. The fluorescent probe for Zn^2+^ based on CPNs was prepared from AIE fluorophore HDBB molecules with Tb^3+^ ion (Tb-HDBB-CPNs). Interestingly, a fluorescence turn-on was observed in the presence of Zn-HDBB-CPNs after the cation exchange process of Tb-HDBB-CPNs with zinc (II). Tb-HDBB-CPNs and Zn-HDBB-CPNs emit different wavelength fluorescence in aqueous solution upon excitation; therefore, the system acts as a ratiometric fluorescent sensor linearly dependent on zinc (II) concentration, from 100 nM to 60 µM.

Yuming Huang and coworkers in 2018 [143] reported an Au nanocluster (AuNC) detecting zinc (II) by AIE effect. Glutathione capped AuNCs (GSH-AuNCs) was synthesised by reduction of Au^3+^ by glutathione. Fluorescence enhance GSH-AuNCs upon addition of 2-methylimidazole in the presence of zinc (II) ion was attributed to the formation of the Zn-MOF (PLQY = 36.6%, about nine times that of AuNCs). GSH-AuNCs resulted in an AIE active sensor that was reported as zinc (II) with a linear range from 12.3 nM to 24.6 mM and LOD = 6 nM. Interestingly, the nanoscaled sensor was successfully applied to assay the content of zinc in human serum, water, milk, and zinc sulfate syrup oral solution samples.

### 4.2. Sensing by Molecular Zn AIEgens 

**Figure 7:**
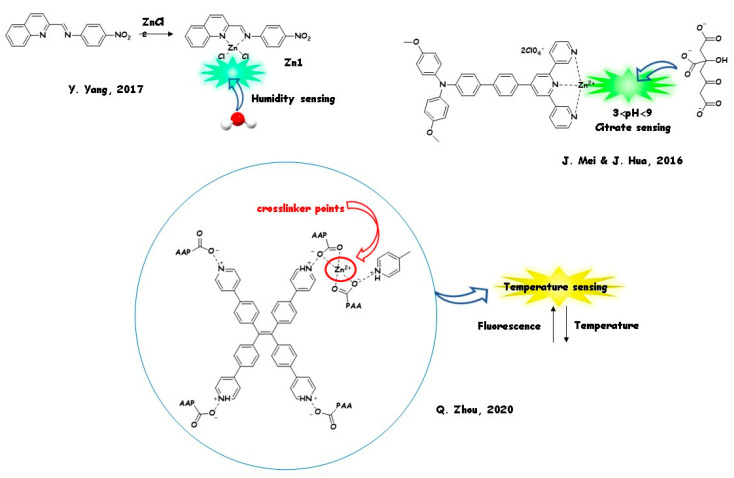
Relevant examples of zinc (II)-based AIE active sensors.

Integrates Zn-containing molecular chemosensors will be discussed in this section, with a focus on the sensing mechanism (Figure 7). As will be detailed described, sensing can take place through an alternation of complexation–decomplexation equilibria involving zinc (II) ions.

Ju Mei and Jianli Hua and coworkers in 2016 [144] developed a new citrate-sensitive, highly selective fluorescence chemosensor (DTPA-TPY-Zn) by integrating an AIE moiety (DTPA-TPY, where TPY guarantees the AIE behaviour) with zinc (II) ion. The probe detects citrate by an immediate significant fluorescence enhancement and LOD = 3.5 × 10^−7^ M. The fluorescence turn-on mechanism is ascribed to the complexation of DTPA-TPY-Zn. Specifically, after coordination to zinc (II) ion, the sensor DTPA-TPY undergoes a decrease in fluorescence emission, but after citrate coordinates to the metal cation of DTPA-TPY-Zn, the derived aggregate state in aqueous solution promotes a fluorescence recovery. The effect of pH on the fluorescence of DTPA-TPY-Zn was investigated, finding a stable response in the pH range from 3 to 9. As citrate is a vital metabolite in the Krebs cycle of aerobic cells, its determination is of biological interest. A quantification of citrate in the artificial urine was successfully performed, proving the potential of DTPA-TPY-Zn in real biological samples. The next year, Yulin Yang and coworkers [145] developed several sensors for the detection of the molecule of life: water. Six novel Schiff-base Zn(II) complexes were synthesised, which exhibit sensing ability in water detection. Remarkable characteristics are the wide linear range in water traces sensing (most can reach 0−94%, *v*/*v*), LODs (0.2%, *v*/*v*), and fast response time (8 s) in various organic solvents. In addition, the sensors demonstrated their application for humidity (42−80%) detection. A mechanism was proposed and validated by experimental and theoretical analysis. Thanks to the water-activated, hydrogen-bonding crosslinking AIE mechanism (WHCAIE), the six zinc (II) complexes bearing chlorine groups/nitro groups can interact with water through intermolecular hydrogen bonding, forming cross-linking networks in the presence of water. The resulting water-activated aggregates of Zn(II) complexes are AIE moieties, whit a superior performance gained by Zn1 (see Figure 7).

An engineered supramolecular system with a key role for zinc (II) ion was designed by Qiong Zhou and coworkers in 2020 [146]. An AIE undergoing hydrogel consisting of a supramolecular network with zinc (II) crosslinking nodes was found to be temperature responsive. Due to the RIM effect in the polymeric segments and the increase of ionic interactions, the hydrogel undergoes a blue shift and an enhancement in fluorescence emission as the temperature decreases.

### 4.3. Sensing by AIE Active Zn-Nanoprobes and Zn-MOFs

We previously described some zinc-based LMOFs as fascinating materials with structural and spectral tunability. The AIE effect has been revealed as a new route for LMOFs fabrication for a wide range of application areas. In contrast to the ACQ effect, AIE-based MOFs display poor emission in dilute solution and higher fluorescence in the aggregated states due to RIM effects undergoing the organic frameworks. LMOFs, where the AIE channel can be selectively activated by a specific analyte, are powerful tools in chemo and biosensing. AIE Zn-MOFs and other nanosized zinc probes are good candidates for sensing of targeted analytes due to the unique vehiculation and the specificity of the porous architectures achievable by zinc (II) cation. Usually, such probes are formed by the reaction of AIE organic ligands and zinc (II). In addition, the replacement of classical heavy metals—often toxic and hardly disposable—with zinc (II) makes Zn AIE nanoprobes sustainable biocompatible tools.

**Figure 8:**
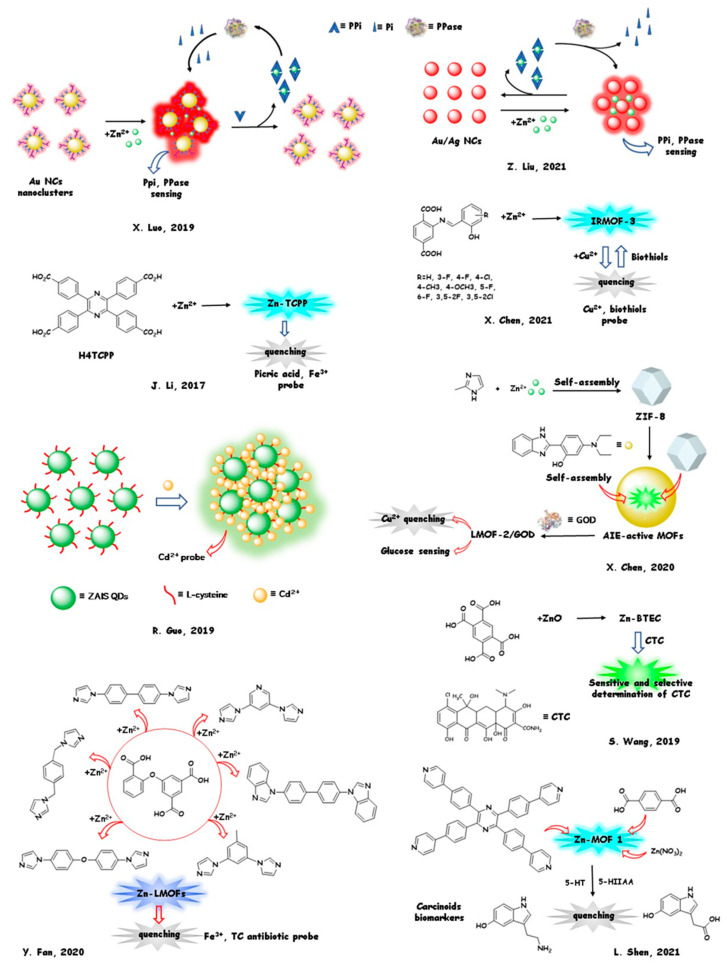
Structures and sensing mode of relevant nanosized Zn-AIEgens.

Selected examples of such nanosized probes will be reported in this section, based on their mono- or multifunctional abilities, starting from chemical targets to biological analytes. Their structures and sensing mode are summarised in Figure 8.

Xiliang Luo and coworkers 2019 [147] produced a sensitive method for the detection of inorganic pyrophosphate (PPi) and pyrophosphatase activity (PPase), which play vital roles in biological systems. Dual-ligand functionalised Au NCs nanoclusters (NCs, dimension between 1nm and 100 nm) exhibiting AIE characteristics were used for fluorescence detection of PPi and PPase, based on a Zn(II)-regulated strategy. Very recently, Zhihong Liu and coworkers [148] developed a similar method for the detection of PPi and PPase. Glutathione stabilised water-soluble alloy Au/Ag nanoclusters (NCs, dimension between 1 nm and 100 nm) exhibiting AIE characteristics were used, still based on a Zn(II)-regulated strategy. The on–off–on mechanism employed in both cases involves the aggregation of Au NCs (or Au/Ag NCs) in the presence of zinc (II) due to electrostatic interaction between Zn^2+^ and the NCs. The fluorescence enhancement is due to the activation of RIR and RIV channels. PPi, containing two phosphate groups, competitively coordinates to zinc (II) ion, subtracting the metal to the previous NCs aggregates. Therefore, a decrease in emission (turn-off) is expected, and the reduction of PL emission can be used to determine the concentration of PPi. On the other hand, PPase catalyzes the hydrolysis of PPi to a single phosphate group and hence break down the PPi-Zn^2+^ non-emissive aggregates. By re-forming of NCs aggregates with zinc (II), the emission turns on. A relationship between the recovery of fluorescence intensity and the amount of PPase leads to a quantitative analysis of PPase activity in water.

Jiyang Li and coworkers in 2017 [149] produced a new rigid symmetric tetracarboxylic ligand 2,3,5,6-tetrakis(4-carboxyphenyl)pyrazine (H4TCPP) with AIE properties as a ligand for building the multifunctional Zn-TCPP MOF. The three-dimensional channel structure of Zn-TCPP displays bright blue luminescence in the presence of CO_2_ and a quenching effect in the presence of picric acid and Fe^3+^ ions. A micrometre-sized phase of Zn-TCPP (named “Zn-TCPP”) exhibiting the best sensing performance was obtained by the solvothermal reaction of H4TCPP and Zn(NO_3_)_2_·6H_2_O. Recently, Xiaoqing Chen and coworkers [150] produced a series of functional IRMOF-3 frameworks with solid-state luminescence and tuneable light emission (from 490 to 608 nm). The MOFs were obtained by reaction of AIE-active Schiff-base ligands with zinc. The AIE-active ligands formed a “flower-like” morphology due to the formation of J-aggregates. A coordination site of the precursors was designed to bind copper (II) ions. IRMOFs were found PL responsive toward Cu(II) and thiols.

Suhua Wang and coworkers in 2019 [151] developed a Zn-LMOF of pyromellitic acid (Zn-BTEC) used for selective and sensitive AIE detection of chlortetracycline (CTC), a broad-spectrum antibiotic, with LOD = 28 nM. CTC molecules defusing into the rigid MOF structure produce additional aggregation leading to a fluorescence enhancement. Zn-BTEC sensors were successfully applied for the sensitive and selective determination of CTC in fish and urine samples. In 2020, Yuhua Fan and coworkers [152] prepared six Zn-LMOFs, starting from flexible or semiflexible moieties (i.e., 5-(2-carboxylphenoxy)isophthalic acid, 11,4-bis(imidazol-l-ylmethyl)benzene, 4,4-bis(imidazolyl)diphenyl ether) combined with rigid moieties (i.e., 4,4-bis(benzoimidazo-1-ly)biphenyl, 4,4-bis(imidazolyl)biphenyl, bis(1-imidazoly)pyridine, and 11,3-bis(l-imidazoly)toluene). The Zn-LMOFs gave a fluorescence quenching sensing response towards Fe(III) ions and TC antibiotic with LOD = 0.35 mM and 0.15 mM, respectively.

Xiaoqing Chen and coworkers in 2020 [153] reported a series of AIE-active LMOFs with good photostability and excellent dispersibility. The emission of the LMOFs could be tuned by varying the substituents on AIEgens. A MOF of the series was used as a fluorescent probe for detecting copper (II) ions in a wide concentration range (1–100 nM) with LOD = 550 pM and for on-site determination of glucose in real serum samples. Recently, Liang Shen and coworkers [154] produced a non-interpenetrated three-dimensional tetraphenylpyrazine (TPP)-based LMOF (denoted as Zn-MOF1) starting from TPyTPP = tetrakis(4-(pyridin-4-yl)phenyl)pyrazine and H2BDC = 1,4-benzenedicarboxylic acid. Zn-MOF1 displays excellent luminescence-quenching sensitivity toward 5-hydroxytryptamine (5-HT) and 5-hydroxyindole-3-acetic acid (5-HIAA) with LODs of 0.50 μM for 5-HT and 0.57 μM for 5-HIAA. Zn-MOF1 performs as efficient sensing of carcinoid biomarkers.

Quantum dots (QDs) are semiconductor nanoparticles with specific optical and electronic properties with the ability to produce PL emission. Ruiqian Guo and coworkers in 2019 [155] designed a nanosized fluorescence probe for Cd^2+^ based on the AIE active QDs containing several inorganic elements, including zinc (ZAIS QDs, i.e., Zn–Ag–In–S QDs). ZAIS QDs were synthesised starting from L-cysteine ligands with an average size of 3.2 nm and an emission peak at 520 nm. The probe was able to detect cadmium (II) ion with LOD = 1.56 mM by activation of the AIE channel due to the interaction between Cd^2+^ and thiol groups on the surface of QDs, which in turn causes electrostatic aggregation. A year later, Jiye Jin and coworkers [156] produced CdSe/ZnS-based QDs applied as ECL (electrochemiluminescence) hydro-phobic dyes acting as AIE active fluorophores for the determination of H_2_O_2_ with LOD = 9.2 × 10^−8^ M.

## 5. Zn AIEgens for Cell Imaging

The scientific research about sensitive and selective fluorescent probes opens new horizons for biological parameters detection. Nowadays, non-invasive and in situ operating biosensors for analysis in living cells are highly required. By modern microscopy and imaging techniques in combination with fluorescence sensor, sharper cytologic details can be visualised and recorded. Cell organisation can be analysed with clarity and precision by employin the sensing tool combined with confocal microscopy and computer integration in a three-dimensional space. From the first fluorescence microscope (due to Heimstadt in 1911 and Lehmann in 1913) to modern bioimaging techniques, more than a century has passed, and research made great strides from the early fluorophores (as rhodamine, fluorescein, flavin derivatives) to the modern highly engineered fluorogenic dyes. Several fluorescent dyes for staining live cells, labelling subcellular organelles, live cell tracking, and carrying in live cells are now commercially available. Nonetheless, the search for new marking systems is constantly evolving. Fluorescence bioimaging continuously requires new real-time tools for bioanalysis and molecular dynamic biological processes with high sensitivity, flexibility, and biocompatibility.

Time-resolved imaging is a modern microscopy technique whereby fast kinetic and PL decay parameters (decay times and the corresponding resolved amplitudes) are directly and simultaneously measured throughout an image in an optical microscope. Thus far, most fluorescent dyes utilised in sensing commonly suffer from severe concentration or the ACQ effect, and their fluorescence lifetimes can be shortened to nanoseconds in the aggregate phase. This behaviour is unwelcomed for the time-resolved fluorescence imaging technique. The employ of AIE active sensors or markers can solve the ACQ problems due to the high fluorescence of the aggregates in aqueous media and short fluorescence lifetimes.

In this context, Zn AIEgens have drawn considerable attention, thanks to the synthetic feasibility, tuneable PL pattern, and biocompatibility.

**Figure 9:**
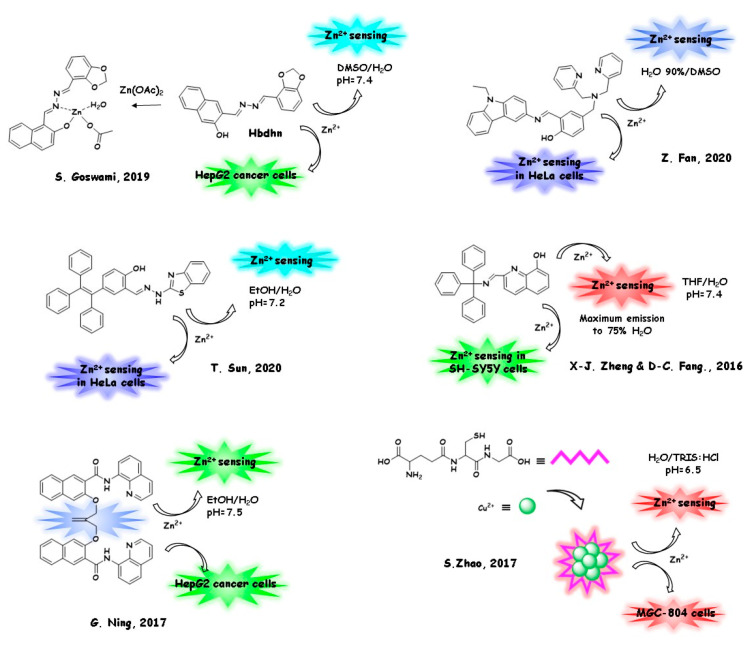
Structures and sensing/marking pattern of relevant AIEgens for zinc (II) detection in living cells.

Again, the involvement of zinc (II) cation with nanosized AIE sensors can be as a biologically relevant analyte or as a part of the sensor itself. In the next section (Section 5.1), we will discuss the monitoring of the zinc (II) level in living biological substrates. The structures and the sensing/marking mechanism are reported in Figure 9.

**Figure 10:**
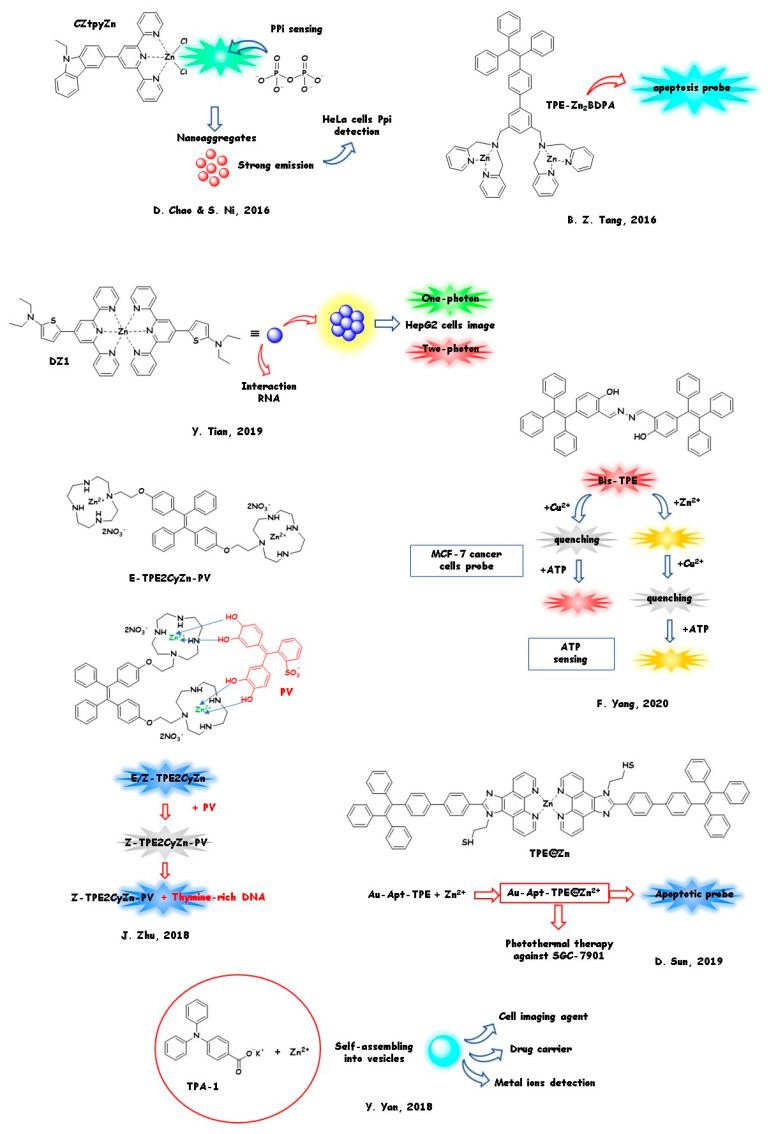
Structures and sensing/marking pattern of zinc-based AIE active biosensors/markers.

In Section 5.2, we will report a selection of the most relevant biosensors and markers containing zinc in their architecture and employed for their sensing/marking ability in living cells (Figure 10). The latter bio-probes can be considered as a class of innovative and desirable biocompatible tools, with a strong technological impact on diagnostic imaging methods.

### 5.1. AIEgens for Zinc (II) Detection in Living Cells 

Even for in vivo tests, probes based on Schiff-base moieties give efficient and selective responses. Sanchita Goswami and coworkers in 2019 [157] reported a Schiff-base chemosensor 1-(benzo(1,3)dioxol-4-ylmethylene-hydrazonomethyl)-naphthalen-2-ol (Hbdhn). The ligand is a zinc (II)-responsive, long-lived fluorescence AIEgen. The sensor was also checked by test paper. A photographic image of Hbdhn on a paper strip in the absence and presence of Zn^2+^ in natural light and under UV light (λ_ex_ = 366 nm) evidenced a potential employment of the sensor in the solid state. Moreover, high efficiency of Hbdhn to monitor zinc (II) cation under physiological conditions was evaluated by fluorescence images of HepG2 cancer cells, detecting a colorimetric turn-on of the fluorescence response in the presence of zinc (II) ion. Zhefeng Fan and coworkers in 2020 [158] synthesised a Schiff-base-chelating probe containing di(2-picolyl)amine (DPA) acting as an AIE chelating agent for Zn^2+^. The probe shows good biocompatibility and a large Stokes shift upon coordination with naked-eye perceivable emission colour. Again, a probe-based test filter paper evidenced a good naked-eye response onto a solid support. A good linear dependence in fluorescence increase was detected between 0.2 and 18 μM, and LOD was 1.3 × 10^−7^ M. The low toxicity and the excellent membrane permeability enable the probe for the application in confocal fluorescence images of HeLa cells. Tongming Sun and coworkers in 2020 [159] produced an AIE probe based on a hydrazonoic skeleton, able to detect Zn^2+^ with LOD = 2.1 × 10^−8^ M and highly sensitive in a pH range of 4.8–12.0. The probe L obtained by condensation of 4-hydroxy-5-aldehyde tetrastyrene and 2-hydrazine benzothiazole, activates by blocking of PET process. It shows good membrane permeability and low cytotoxicity and can be applied in HeLa living cells for endogenous and exogenous zinc (II) detection.

Among the fluorescent AIE probes developed in recent years, quinoline-based probes attracted the interest of researchers due to their strong binding ability towards metal cations and excellent fluorescent properties [160]. A quinoline derivative was employed by Xiang-Jun Zheng and De-Cai Fang and coworkers 2016 [161]. They explored the PL properties of the zinc (II) complex obtained from 2-(trityliminomethyl)-quinolin-8-ol ligand (l) acting as a *N*,*O* pincer. ZnL_2_ units formed stacking by the coordination bonds and intermolecular π–π interactions (J-aggregates). The ligands show no fluorescence, and the complex is emissive in the aggregate phase due to the restriction of the ESIPT process. Therefore, ligand L is an ESIPT-based AIE chemosensor for Zn(II) in an aqueous medium. To prove the ability of the ligand to detect Zn^2+^ in living cells, bioimaging experiments were carried out in SH-SY5Y cells incubated with L 20μM for 16h and treated with perchloride zinc (II) salt 20 μM for 30 min, recording a bright green fluorescence when complex formed. In 2017 Guiling Ning and coworkers [162] synthesised two organic AIE active fluorophores containing bis-naphthylamide and quinoline moieties. An isobutylene group was introduced in one case to increase rigidity and to reduce intramolecular molecular rotations (RIR). As expected, the more encumbered ligand shows a higher fluorescence response towards Zn^2+^. The probe exhibits a highly ratiometric fluorescence response towards zinc (II) ion and excellent cell permeability in human hepatoma cancer cells HepG2, with a binding constant of 5 × 10^−7^ M^−1^ and LOD = 2.6 nM.

Glutathione (GSH)-capped nanoclusters were reported in Section 4 for their sensing ability. In 2017, Shulin Zhao and coworkers [163] prepared glutathione (GSH)-capped copper nanoclusters (CuNCs) with red emission for Zn^2+^ detecting and explored their potential by confocal fluorescence images in MGC-803 cells. The AIE mechanism was based on zinc (II) triggered aggregation of CuNCs, inducing the increase of PLQY from 1.3% to 6.2%. The probe showed a fast response in the detection range from 4.68 to 2240 μM and LOD = 1.17 μM.

### 5.2. Zn AIEgens for Biological Targets in Living Cells

TPY, as a *N*,*N*,*N*-tridentate ligand able to form stable complexes with zinc (II), is the building block of several zinc-based AIE biological probes. In 2016, Duobin Chao and Shitan Ni [164] developed two TPY-based Zn(II) complexes for selective nanomolar PPi detection in a water medium, with LOD = 5.37 nM. The sensors are based on AIE and ICT effects due to the formation of nanoaggregates. The probes were successfully employed for nucleus staining in living cells. Specifically, confocal fluorescence images of HeLa cells incubated with the probes were recorded, showing potential applications in PPi detection by cell imaging and diagnosis. In 2019 Yupeng Tian and coworkers [165] presented a multiphoton bioimaging TPY-based zinc (II) complex bearing a thiophene bridge (DZ1). Due to the AIE effect, the probe emits bright yellow-green fluorescence under physiological conditions. DZ1 showed an excellent response to RNA, compared with commercially multiphoton probes, due to the self-aggregation ability under physiological conditions and excellent photostability. The spectral changes of DZ1 in binding RNA result in a twofold enhancement of the three-photon action cross-sections located at the second near-infrared window (1700 nm). Fluorescence microscopy images of HeLa cells stained with different concentrations of DZ1 (1.0–10 mM) and in aqueous phosphate buffered were presented.

The apoptosis level plays a crucial role in cell growth, and its dysregulation may result in cancers and neurodegenerative diseases. In 2016, Ben Zhong Tang and coworkers [166] synthesised a novel AIE-active fluorescent probe (TPE–Zn_2_BDPA) comprised of two components: tetraphenylethene (TPE) as the AIEgen, and 2,6-bis(zinc (II)-dipicolylamine)phenoxide (Zn_2_BDPA) as the targeting group for phosphatidylserine (PS). PS has emerged as an attractive indicator for apoptosis detection. It shows fluorescence response to apoptotic cells and differentiates the early and late stages of cell apoptosis. Light-blue emissive TPE–Zn_2_BDPA with low cytotoxicity and excellent biocompatibility resulted in being comparable with two commonly used probes for apoptosis detection (Annexin V-FITC and propidium iodide, PI) commonly employed to image HeLa and HepG2 cells. Fafu Yang and coworkers in 2020 [167] synthesised an AIE probe based on a hydrazono-bis-tetraphenylethylene (Bis-TPE) multiple-detecting adenosine triphosphate (ATP) and two biologically relevant metal cations. Bis-TPE exhibited good AIE fluorescence at 550–700 nm based on the highly conjugated electron effect. The probe display sensing abilities for copper (II) ion in a fluorescence turn-off mode. The strong red fluorescence in the Bis-TPE system, quenched by Cu^2+^, can be recovered by adding ATP. On the other hand, the addition of zinc (II) to Bis-TPE produces a relevant blue shift, and the orange fluorescence of the Bis-TPE-Zn^2+^ system can be quenched by copper (II) and then recovered by adding ATP. The “allochroic off–on” orange fluorescence in Bis-TPE-Zn^2+^-Cu^2+^ combined system was applied to sense copper (II) and zinc (II) cations and ATP both on test paper and by confocal fluorescence images of MCF-7 cancer cells.

Jintao Zhu and coworkers in 2018 [168] developed a metallo–supramolecular assembly (Z/E-TPE2CyZn-PV) consisting of a tetraphenylethene (TPE)-based dinuclear Zn^2+^–cyclen complex and pyrocatechol violet (PV). The PV fragment can bind with TPE2CyZn and quenched the background fluorescence. Z/E-TPE2CyZn-PV acted as a fluorescence turn-on chemosensor for selective detection of the T-rich nucleic acid in aqueous buffers, discriminating the T-rich single-stranded DNA (ssDNA) from double-stranded DNA (dsDNA). Z-TPE2CyZn-PV proved to be an indicator displacement assay (IDAs) inside living cells by fluorescence microscopy images of B16–F10 cells.

As reported in previous sections, nanoprobes found their ultimate application in luminescence sensing. Detection of biological species in physiological conditions by employing nanoparticles are drawing more and more attention by virtue of their selectivity, low cytotoxicity, and cell permeability. Multifunctional nanosized probes for cell imaging, drug-carrying, and metal ion detection are effective cutting-edge in scientific research. Recently, hybrid organic–inorganic nanoprobes based on the bio-friendly zinc (II) cation [169,170,171,172,173] have been explored as highly engineered sensors and markers for bioimaging [63,126]. Rare articles have been produced in which the link between the AIE properties and the sensing ability has been highlighted and explored.

Dongdong Sun and coworkers in 2019 [174] fabricated dual-targeted gold nanoprisms, whereby bare gold nanoprisms (AuNPR) were conjugated to phenanthroline derivatives tetraphenylethene (TPE) functionalised and exhibiting AIE characteristics. The probe was further stabilised with target peptide aptamers via Au-S bonds (Au-Apt-TPE). The free nitrogen atoms in Au-Apt-TPE can chelate zinc (II) ions (obtaining Au-Apt-TPE@Zn) which were used for selectively monitoring early stage apoptotic cells. Remarkably, Au-Apt-TPE@Zn under NIR irradiation showed effective penetration and photothermal therapy against SGC-7901 human gastric carcinoma cells growth. In vivo studies revealed deep penetration of Au-Apt-TPE@Zn under NIR irradiation and dual-model imaging application for cancer-targeted fluorescence imaging. In 2018, Yun Yan and coworkers [175] reported a metallo–organic fluorescent vesicle based on the triarylamine carboxylate AIE molecules (TPA-1). When zinc (II) ions electrostatically interact with the TPA-1 molecule, the complex Zn(TPA-1)_2_ forms, which further self-assembles into vesicles. TPA-1 molecules confined in the vesicle membrane display AIE behaviour. The vesicles are excellent cell imaging agents (as tested in Hela cells) by two-photon emission. The probes can be a powerful tool for cell imaging, drug-carrying, and metal ion detection. 

## 6. Conclusions

The review briefly summarised the development history, the AIE-channel activation, and the recent achievements of Zn AIEgens research. During the past 10 years, the development in AIEgens research has undergone rapid growth. Novel AIE active systems have been designed and synthesised; their properties and mechanism have been deeply investigated by experimental verification and theoretical models. The basic study gave the boost to novel AIE systems for more and more applications, from optics to sensing and bioimaging. The search for hybrid inorganic–organic compounds with unique photophysical properties is crucial in developing novel AIEgens. In this perspective, the abundant, inexpensive, and nontoxic zinc (II) metal cation offers many opportunities. The benefits of using zinc complexes instead of conventional luminescent AIE active organic materials or in place of other metal cations have been examined. Acting as a “chemical clip”, zinc (II) ion imposes a constraint to the ligand or assembles the ligands in AIE active macro-architectures, with a scarce contribution to the electronic pattern and a large contributor to the RIM effect.

The most relevant examples of Zn AIEgens from the last 5 years (2016–2021) were collected, and the discussion articulated with the level increasing. The structure–properties relationship and the AIE triggered applications were the thread running through the text. In brief, the review displays an overview, from the novel fully characterised Zn AIEgens to the Zn AIEgens involved in chemo and biosensing. As zinc (II) cation is of great importance to humans and other biological systems, the sensing ability of AIEgens toward the metal was a relevant section of our discussion. Finally, we reported applications of Zn AIEgens targeted for living cell imaging as materials designed for the novel technological frontier.

We trust that the comprehensive and informative approach of this review could trigger new ideas because ideas open new synthetic strategies, promote new ways of projecting, and address new avenues for the next generation of lighting and imaging devices.
